# Correction: Copper technology in the Arabah during the Iron Age and the role of the indigenous population in the industry

**DOI:** 10.1371/journal.pone.0346086

**Published:** 2026-03-31

**Authors:** The PLOS One Editors

## Notice of Republication

This article [1] was republished on February 24, 2026 to address an issue identified post-publication. Please download this article again to view the correct version.
